# Brainstem Associated Somatosensory Evoked Potentials and Response to Vagus Nerve Stimulation: An Investigation of the Vagus Afferent Network

**DOI:** 10.3389/fneur.2021.768539

**Published:** 2022-02-18

**Authors:** Hrishikesh Suresh, Karim Mithani, Karanbir Brar, Han Yan, Samuel Strantzas, Mike Vandenberk, Roy Sharma, Ivanna Yau, Christina Go, Elizabeth Pang, Elizabeth Kerr, Ayako Ochi, Hiroshi Otsubo, Puneet Jain, Elizabeth Donner, O. Carter Snead, George M. Ibrahim

**Affiliations:** ^1^Institute of Biomedical Engineering, University of Toronto, Toronto, ON, Canada; ^2^Program in Neuroscience and Mental Health, The Hospital for Sick Children Research Institute, Toronto, ON, Canada; ^3^Division of Neurosurgery, Department of Surgery, The Hospital for Sick Children, University of Toronto, Toronto, ON, Canada; ^4^Division of General Surgery, Department of Surgery, University of Toronto, Toronto, ON, Canada; ^5^Institute of Health Policy, Management and Evaluation, University of Toronto, Toronto, ON, Canada; ^6^Division of Neurology, The Hospital for Sick Children, Toronto, ON, Canada; ^7^Department of Psychology, The Hospital for Sick Children, Toronto, ON, Canada; ^8^Institute of Medical Science, University of Toronto, Toronto, ON, Canada

**Keywords:** vagus nerve stimulation, somatosensory evoked potentials, epilepsy, outcomes, vagus afferent network

## Abstract

Despite decades of clinical usage, selection of patients with drug resistant epilepsy who are most likely to benefit from vagus nerve stimulation (VNS) remains a challenge. The mechanism of action of VNS is dependent upon afferent brainstem circuitry, which comprises a critical component of the Vagus Afferent Network (VagAN). To evaluate the association between brainstem afferent circuitry and seizure response, we retrospectively collected intraoperative data from sub-cortical recordings of somatosensory evoked potentials (SSEP) in 7 children with focal drug resistant epilepsy who had failed epilepsy surgery and subsequently underwent VNS. Using multivariate linear regression, we demonstrate a robust negative association between SSEP amplitude (*p* < 0.01), and seizure reduction. There was no association between SSEP latency and seizure outcomes. Our findings provide novel insights into the mechanism of VNS and inform our understanding of the importance of brainstem afferent circuitry within the VagAN for seizure responsiveness following VNS.

## Introduction

Epilepsy is amongst the most common and debilitating neurological disorders in children, affecting 1–2% of the pediatric population (1). Nearly 30–40% of patients are resistant to antiepileptic medications, and may benefit from surgical management (2). Vagus nerve stimulation (VNS) is a safe and well-tolerated treatment option for drug-resistant epilepsy (DRE) that involves electrical stimulation of the vagus nerve at the level of the neck using an implantable device.

Seizure outcomes after VNS are highly variable and difficult to predict (3, 4), partly due to the fact that its mechanism of action remains incompletely understood (5). Recent advances in connectomics have identified a number of structural and functional biomarkers associated with VNS response (6, 7). For example, diffusion tensor imaging (DTI) and magnetoencephalography (MEG) investigations have revealed that increased structural and functional connectivity within the vagus afferent network (VagAN)—a complex neuronal network that appears to be engaged during stimulation of the vagus nerve—portends VNS response (5, 6, 8). In particular, VNS responders demonstrate greater engagement of VagAN circuitry with stimulation of the median nerve, which shares overlapping afferent neuronal circuitry with the vagus nerve (8).

The neuromodulatory response of VNS is critically dependent upon afferent brainstem circuitry. Most vagus nerve fibers are comprised of afferent projections to the nucleus tractus solitarius (NTS), which has a wide distribution to various areas of the brainstem involved in modulating forebrain activity (9). Several of these direct and indirect projections in this region include the noradrenergic locus coeruleus (LC), the serotonergic dorsal raphe nucleus (DRN), and the parabrachial nucleus (PBN). Despite increasing evidence pointing to intrinsic brain network differences in responders to VNS, relative to non-responders, there have been little data to date that elucidate the role of brainstem pathways in mediating VNS responsiveness (10). Given the critical role of the brainstem pathways within the VagAN (5, 11), we sought to study the robustness of brainstem pathways and their association with VNS response.

In the current study, we explore the association between characteristics of the subcortically recorded component of the somatosensory evoked potential (SSEP) related to brainstem function and their association with VNS response. Previous studies have leveraged the overlapping circuitry between the spinothalamic tract and vagus afferent pathway at the level of the ventral posterolateral and ventral posteromedial nuclei of the thalamus, respectively, to identify differences in cortical activations in response to median nerve stimulation in responders compared to non-responders (8). Here, we index brainstem pathway robustness by the brainstem associated SSEP latency and amplitude following bilateral ulnar nerve stimulation in a cohort of children with drug resistant epilepsy undergoing implantation of a VNS device. We hypothesize that differences in brainstem associated evoked responses are associated with seizure response following VNS. The current work provides insights into the critical role of the brainstem pathways within the VagAN and form the basis for future work aimed at presurgically identifying ideal candidates for VNS.

## Methods

### Patient Selection

We performed a retrospective cohort study of 7 pediatric patients who previously underwent epilepsy surgery prior to VNS, during the implantation of which, intraoperative monitoring with ulnar nerve SSEP was performed. SSEP were recorded under general anesthesia. Demographic information for subjects is included in Table 1. This study complies with the principles outlined in the Declaration of Helsinki and was approved by the Research Ethics Board at The Hospital for Sick Children.

**Table 1:** Demographic information of included patients.

**Characteristic**	**Overall (*n =* 7)**
Median age, years (range)	12.3 (9.1–18.0)
**Sex**, ***n*** **(%)** Male Female	4 (57.1) 3 (42.9)
Median follow-up, years (range)	1.4 (0.6–7.0)
Median duration of seizures at time of VNS, years (range)	7.1 (2.0–12.0)
Median duration between epilepsy surgery and VNS, years (range)	3 (0.8–7)
Mean number of anti-seizure drugs in treatment regimen (±SD)	2.57 (±0.79)
**Seizure etiology**, ***n*** **(%)** Structural Genetic	4 (57.1) 3 (42.9)
**Previous epilepsy surgery**, ***n*** **(%)**	7 (100.0)
Resection of epileptogenic foci in the Rolandic cortex Lesionectomies Temporal lobectomy Resection of tuber + temporooccipital lobectomy	2 (28.6) 3 (42.9) 1 (14.3) 1 (14.3)
**Reason for surgical failure** Eloquent cortex—limited resection Multifocal disease Biopsy only Unknown	2 (28.6) 2 (28.6) 1 (14.3) 2 (28.6)
**Seizure characteristic** Bilateral tonic-clonic Focal onset	2 (28.6) 5 (71.4)
**Pre-VNS (v)EEG ictal localization** Focal activity Multifocal activity Generalized activity	7 (100.0) 0 (0.0) 0 (0.0)
**Pre-VNS (v)EEG interictal localization**	
Focal activity	4 (57.1)
Multifocal activity	3 (42.9)
**(v)EEG laterality** Left Right Bilateral	5 (71.4) 1 (14.3) 1 (14.3)
**Findings**	
Subependymal nodule (not tuberous sclerosis)	1 (14.3)
Tonsillar ectopia	1 (14.3)
Non-specific T2/FLAIR high signal lesions	2 (28.6)
Tuberous sclerosis	1 (14.3)
Focal cortical dysplasia	2 (28.6)

### Neurophysiologic Investigations

It is routine at our institution for all patients undergoing epilepsy surgery to undergo intraoperative SSEP studies for monitoring purposes (12). Sub-cortical SSEPs from stimulation of the right and left ulnar and nerves were recorded using the Nicolet Endeavor CR platform (Natus Medical, Middleton, WI). Constant current stimulation was provided through pre-gelled surface electrodes (LifeSync Neuro, Lutz, Florida) placed over the ulnar nerve at the wrist. Potentials were elicited using a 300 us square-wave pulse delivered at a rate of 4.7 Hz. Stimulation intensity ranged from 12 to 25 mA for ulnar nerve and was adjusted based on the maximal amplitude response for each individual patient. Sub-cortical potentials were recorded from subdermal needle electrodes placed at the surface of the second cervical vertebra and were referenced to Fpz according to the International 10–20 system (13). Responses were averaged until clear, reproducible waveforms were identified, up to a maximum of 300 trials. Responses were recorded using a 30–500 Hz bandpass filter and waveforms were displayed in a 50-ms window.

### Statistical Analysis

Robust Multivariate linear fixed effects models were generated using MM estimation to analyze the association between percentage reduction in seizure frequency and either subcortical SSEP latency or amplitude. Patient age at the time of VNS implantation and side of stimulation were included as covariates in these models. The analysis was done in R (14) version 4.1.1, and the *robustbase* package (15).

## Results

### Subject Demographics

Seven patients were included in this study with a mean age of 12.3 (9.1–18) years. Four males and 3 females were included. The demographic data are presented in Table 1 along with the seizure response rates. All patients in this study had previously undergone surgery for epilepsy prior to insertion of VNS, but the surgery had failed. Specifically, 2 patients underwent resection of epileptogenic foci in the Rolandic cortex, 3 patients had lesionectomies (2 for focal cortical dysplasia, 1 for tuberous sclerosis, 1 for filaminopathy), 1 patient had a temporal lobectomy, and 1 patient underwent both a tuberectomy and a temporooccipital lobectomy. All patients had recurrence of seizures that warranted implantation of VNS. Median duration of seizures at the time of VNS was 7.1 (2–12) years. Median duration between original epilepsy surgery and VNS was 3 (0.83–7) years. Mean follow up was 1.4 (0.6–7) years.

### Seizure Characteristics and Localization

The majority of patients had exclusively focal seizures (71%), while 28% had bilateral tonic-clonic seizures. On average, patients were on 2.57 ± 0.79 antiseizure medications.

Five patients were found to have focal ictal patterns on preoperative EEG. Interictal activity was found to be focal in four patients, and multi focal in 2 patients. None of the patients demonstrated generalized ictal activity, however, one patient demonstrated diffuse interictal activity.

Two patients underwent incomplete resections due to the pathology being in eloquent cortex, two other patients were found to have multifocal disease, and one patient only underwent a biopsy. The remaining two patients had seizure recurrence despite having complete resections. However, because they did not have EEGs before VNS, the reason for failure of the previous procedure is unclear.

### Imaging Findings

Apart from previous postsurgical findings, one patient had nonspecific T2 changes, one had tonsillar ectopia, and one had a subependymal nodule, two had focal cortical dysplasia and one has tuberous sclerosis.

### SSEP Correlation With Surgical Outcome

**Figure 1:**
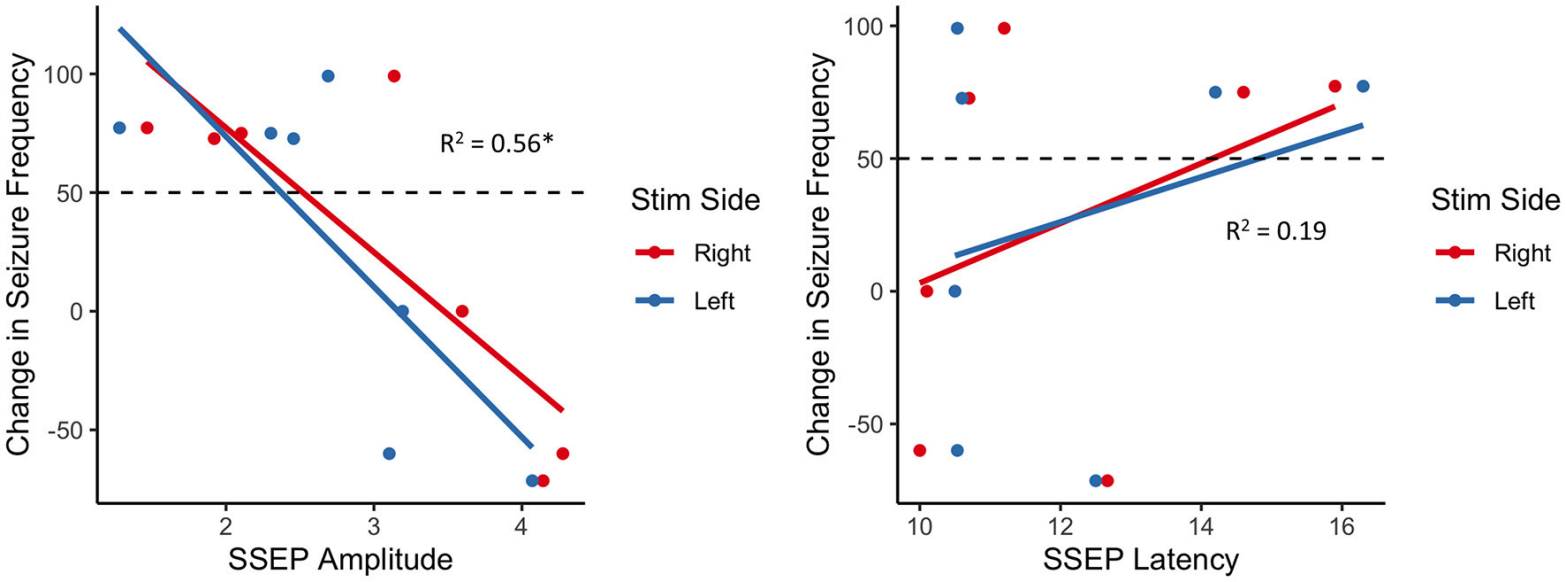
Association between change in seizure frequency and brainstem SSEP amplitude (left) and latency (right); there is a robust negative relationship between SSEP amplitude and change in seizure frequency, with responders (above dashed line) generally exhibiting lower amplitudes than non-responders (below dashed line); *p < 0.01.

Considering seizure reduction as a continuous outcome, robust generalized linear regression models were employed to identify associations between SSEP properties and VNS response, while adjusting for the child's age. We found a statistically significant negative association between SSEP amplitude and percent reduction in seizure frequency (β = −59.3, adjusted *R*^2^ = 0.57, *p* = 7.67 × 10^−7^), with no significant effect of age (*p* = 0.25) or side of stimulation (*p* = 0.70) (Figure 1). Conversely, there was no significant association between change in seizure frequency and SSEP latency.

## Discussion

Vagus nerve stimulation is a promising surgical intervention for certain patients with DRE. Nevertheless, heterogeneous outcomes following surgery underscore the need for preoperative biomarkers to inform patient selection. Intrinsic brain differences within the VagAN between responders and non-responders to VNS are promising biomarkers to predict responsiveness to therapy (16).

One region of the VagAN that has yet to be extensively studied is the brainstem afferent circuitry. Although evoked potentials could be measured from VNS—either transcutaneous or at the time of surgical implantations, technical challenges, such as contamination of signals due to artifacts from neck muscle activation (17) render direct analysis of evoked responses from the vagus nerve impractical. Given the overlapping circuitry between the spinothalamic tract and vagus afferent pathways (8), we sought instead, to assess the utility of SSEPs associated with brainstem function with ulnar nerve stimulation to identify the association between brainstem afferent circuitry and VNS response. We identified a robust negative association between brainstem SSEP amplitudes and changes in seizure frequency with lower amplitudes associated with better response to VNS.

Previous studies have shown that changes in SSEP amplitude, latency, and/or absence or presence of certain SSEP components can be indicative of aberrant CNS connectivity (18, 19). For example, patients with unilateral cerebrovascular lesions have abnormal, high-amplitude SSEPs over the non-affected hemisphere (20). A higher SSEP amplitude in the cerebral cortex or brainstem can also be a marker of increased cortical excitability and reduced seizure threshold. For example, children with various neurological disorders, including several forms of epilepsy, frequently exhibit larger amplitudes of cortical SSEPs (21–23). In particular, patients with progressive myoclonic epilepsy and cortical myoclonus show characteristic “giant” SSEPs, indicating that patients with lower seizure thresholds often have corresponding aberrant SSEP readings (24–27). Patients with systemic illnesses with known CNS involvement, such as primary Sjögren's syndrome, also frequently exhibit increased SSEP amplitudes compared to healthy controls (28).

Our results taken in the context of prior findings present two possibilities. The first is that increased brainstem SSEP amplitudes are indicative of increased cortical disease burden, related to a lower seizure threshold, and a decreased susceptibility to VNS therapy. Alternatively, less robust brainstem circuitry, indexed by increases in subcortical SSEP amplitudes, result in lesser ability of VNS to modulate cortical activity. There was no significant association between SSEP latency and VNS outcome, however we did not correct for limb length intraoperatively. This could be the source of the insignificant result.

## Conclusion and Limitations

VNS is an established treatment for patients with DRE, but there are few biomarkers to inform patient selection. Here, we identify robust negative associations between SSEP amplitude and VNS response. This study is limited by its relatively small sample size, and short period of follow-up. Short term follow up could underestimate the true effect of VNS therapy, and thus affect the strength of the association with brainstem associated SSEPs. The utility of brainstem associated SSEPs for this purpose should be further explored in future studies. Continued neurophysiological investigations on intrinsic nervous system connectivity within the brainstem and its association with VNS treatment response in DRE represent important steps toward both optimizing patient selection and further elucidating the mechanism of action of VNS.

## Data Availability Statement

The raw data supporting the conclusions of this article will be made available by the authors, without undue reservation.

## Ethics Statement

The studies involving human participants were reviewed and approved by Hospital for Sick Children Research Ethics Board. Written informed consent to participate in this study was provided by the participants' legal guardian/next of kin.

## Author Contributions

SS, MV, and RS performed the intraoperative data collection and helped preprocess the data for analysis. AO and HO provided guidance with the analysis of the data. HS and KM analyzed the data. HS, KM, and KB wrote the manuscript with input and feedback from all authors. HY, IY, CG, EP, EK, PJ, ED, and OS provided guidance on the interpretation of the data, and the associated clinical significance. GI was involved along all stages and oversaw the overall direction and planning. All authors contributed to the article and approved the submitted version.

## Conflict of Interest

Unrelated to this study author GI received funds from LivaNova Inc. The authors declare that the research was conducted in the absence of any commercial or financial relationships that could be construed as a potential conflict of interest.

## Publisher's Note

All claims expressed in this article are solely those of the authors and do not necessarily represent those of their affiliated organizations, or those of the publisher, the editors and the reviewers. Any product that may be evaluated in this article, or claim that may be made by its manufacturer, is not guaranteed or endorsed by the publisher.
